# Supplementary material for: AIP56, an AB toxin secreted by Photobacterium damselae subsp. piscicida, has tropism for myeloid cells
Source: Front Immunol. 2025 Jan 13;15:1527088. doi: 10.3389/fimmu.2024.1527088 (PMC11769971; doi:10.3389/fimmu.2024.1527088)
Supplement: Supplementary file 2 [file Image1.pdf]

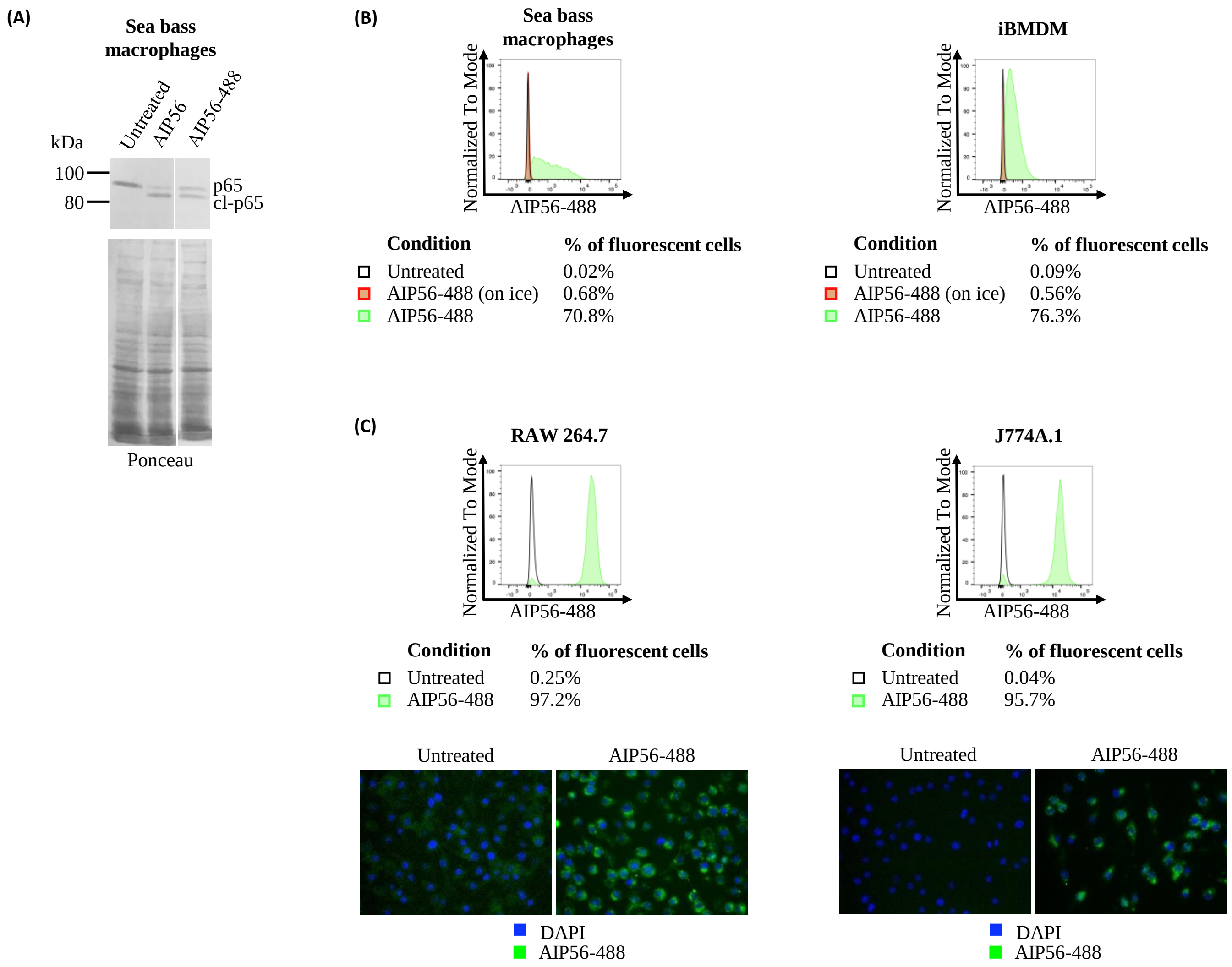

Figure S1. AIP56-488 is successfully internalized by the cells. **(A)** Representative WB analysis of NF-κB p65 cleavage in sea bass macrophages left untreated or incubated with AIP56 or AIP56-488 for 15 min on ice followed by 4 h at 22°C. NF-κB p65 was cleaved by both toxins. **(B)** Sea bass macrophages and immortalized bone marrow derived macrophages (iBMDM) were incubated with AIP56-488 on ice, washed, and either kept on ice to inhibit endocytosis (see e.g., Weigel and Oka 1981 Journal of Biological Chemistry 256:2615-2617. https://doi.org/10.1016/S0021-9258(19)69656-0 and Szewczyk-Roszczenko et al 2023 Cells 12:2312. https://doi.org/10.3390/cells12182312), or transferred to 22ºC for 15 min or to 37ºC for 30 min, for sea bass or mammalian cells, respectively. When analyzed by flow cytometry, AIP56-488-positive cells were only detected in sea bass or mammalian cells incubated at 22ºC or 37ºC, respectively. This confirms that the flow cytometry protocol used only detects cells with endocytosed toxin, in agreement with microscopy results reported in Pereira et al 2014 (Infect Immun 82:5270–5285. doi: 10.1128/IAI.02623-14), showing that in cells incubated with AIP56-488, fluorescence can only be detected after internalization and concentration of AIP56-488 in the endocytic compartment. **(C)** Representative flow cytometry plots and correspondent fluorescence microscopy images of AIP56-488 internalization by mouse RAW 264.7 and J774A.1 cell lines. Cells were left untreated or incubated with AIP56-488 for 15 min on ice followed by 30 min at 37°C. AIP56-488-positive cells were detected by flow cytometry or fluorescence microscopy after staining the nuclei with DAPI (blue).

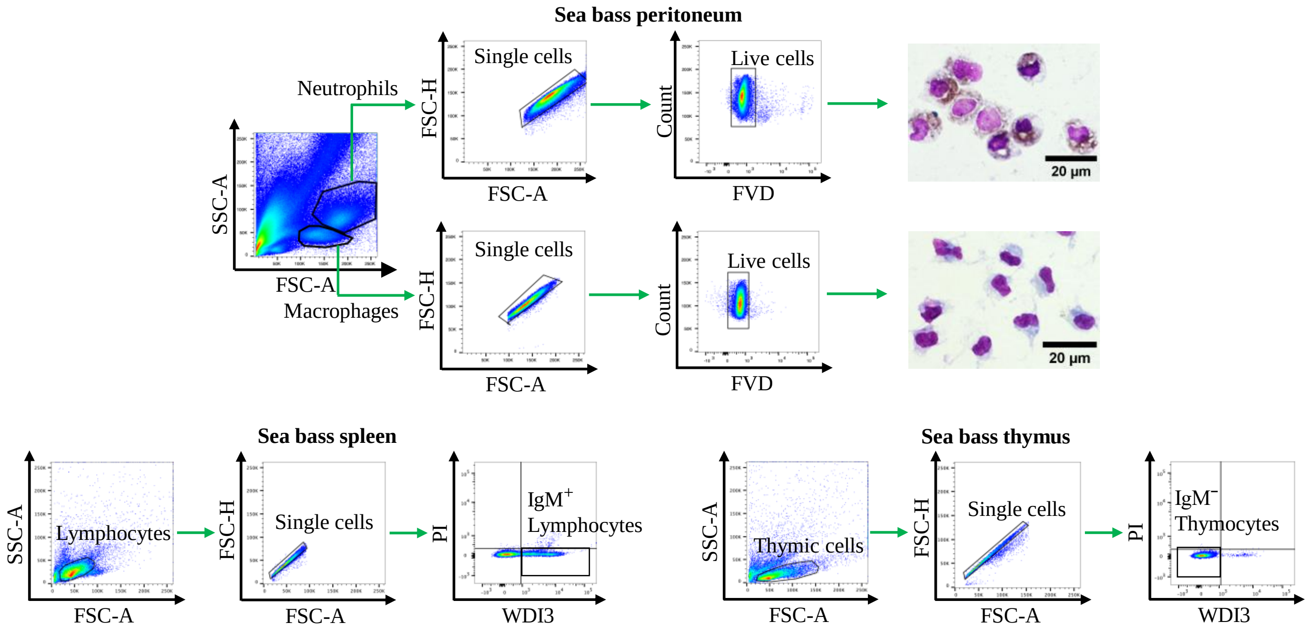

Figure S2. Gating strategy applied to identify sea bass neutrophils and macrophages from peritoneum and IgM^+ lymphocytes (B-cells) and IgM^- cells (putative thymocytes) from sea bass spleen and thymus samples, respectively, after Percoll gradient. Neutrophils were assessed based on their morphology (FSC-A vs SSC-A) considering that the analyzed samples were from inflamed peritoneal cavities after 6 h injection of UV-killed MT1415, where neutrophils are the majority of the granulocytes. Macrophages from the same cavities were also assessed based on FSC-A vs SSC-A and analyzed as control. After selecting viable neutrophils and macrophages, cells were sorted and cytospins were performed to confirm the identity of the selected cells. Hemacolor technique was performed and Antonow was used for peroxidase detection to stain neutrophils (brown), allowing to distinguish between these two cell types. Lymphoid cells were selected based on their morphology (SSC-A vs FSC-A) and IgM expression. Fixable viability dye (FVD) and propidium iodide (PI) were used to identify viable neutrophils/macrophages and lymphoid cells, respectively.

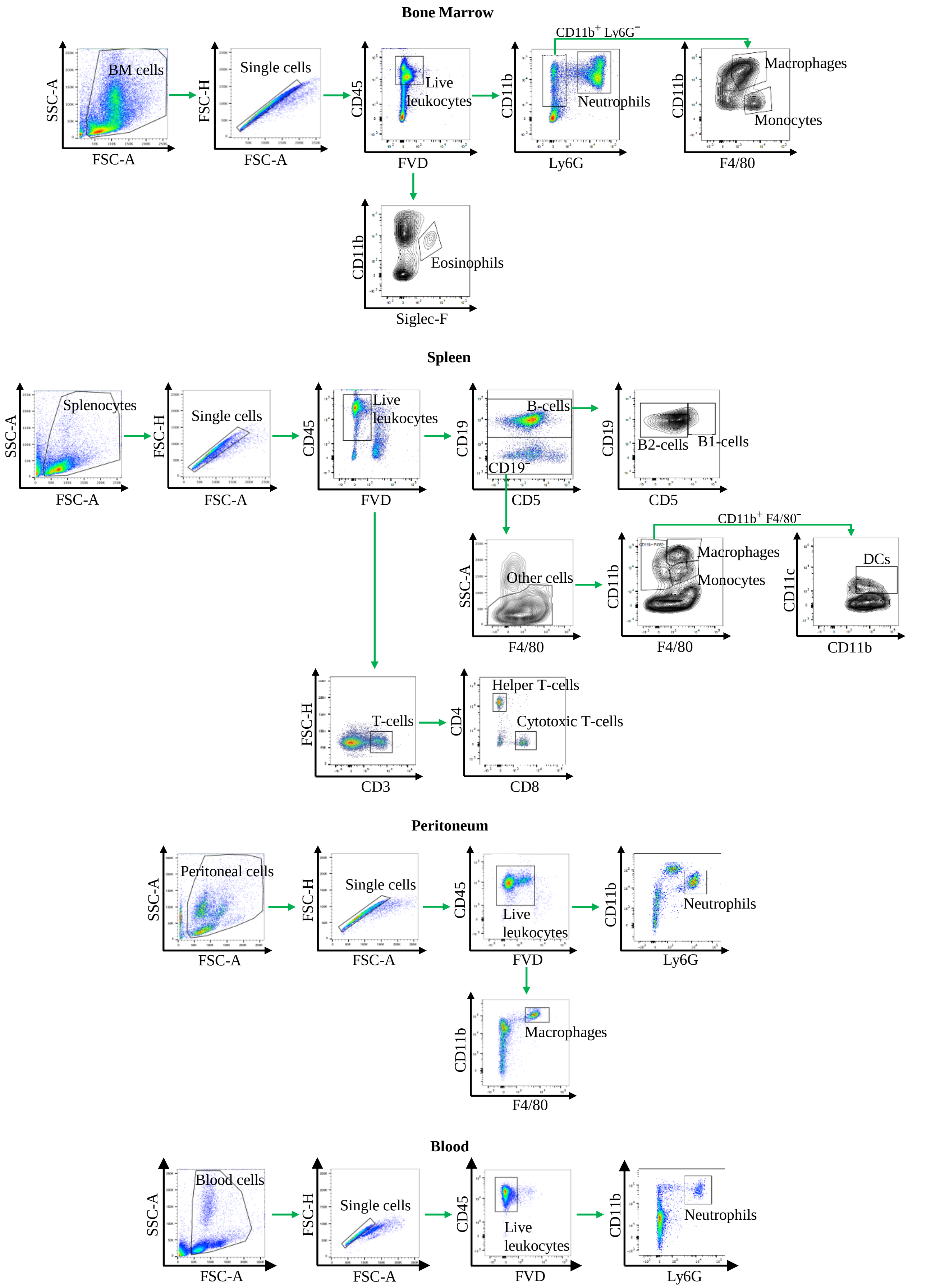

Figure S3. Gating strategy applied to identify different mouse leukocytes from bone marrow (BM), spleen, peritoneum (inflamed with thioglycolate for 6 h) and blood samples, after identifying general cell population by morphological parameters (SSC-A vs FSC-A) and eliminating duplets (FSC-H vs FSC-A).

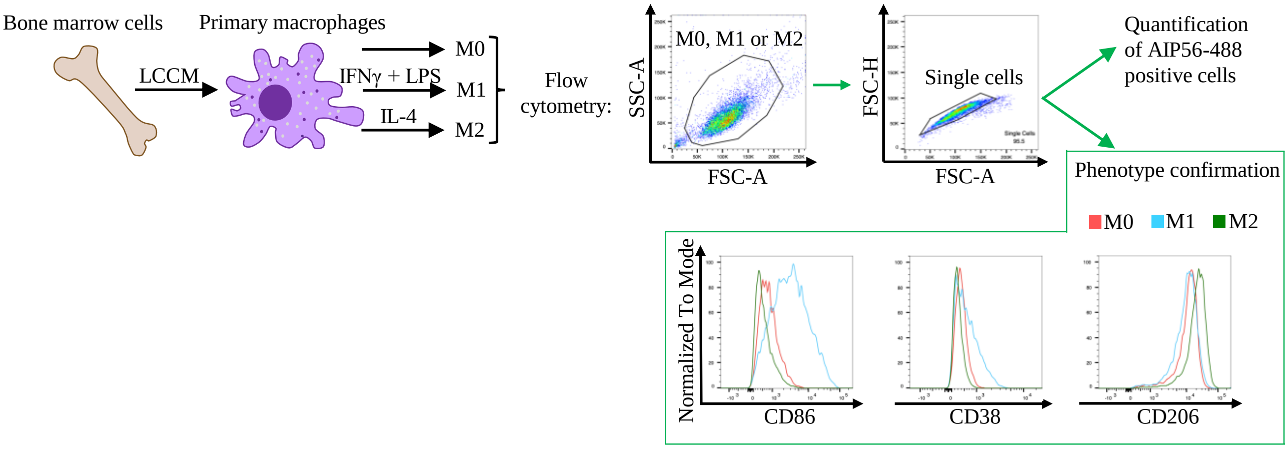

Figure S4. Schematic description of mBMDM differentiation and polarization. Representative plots of the gating strategy applied to confirm M0, M1-like and M2-like phenotype, after identifying general cell population by morphological parameters (SSC-A vs FSC-A) and eliminating duplets (FSC-H vs FSC-A), based on their expression of CD86, CD38 and CD206, respectively.

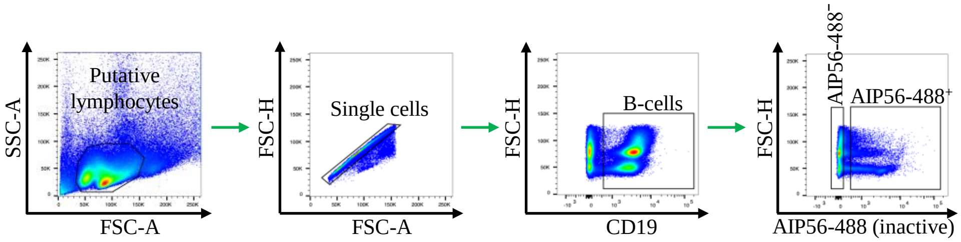

Figure S5. Gating strategy applied to isolate mouse AIP56-488^{+} and AIP56-488^{-} B-cells from spleen, after identifying general cell population by morphological parameters (SSC-A vs FSC-A), eliminating duplets (FSC-H vs FSC-A) and selecting B-cells (CD19^{+}).

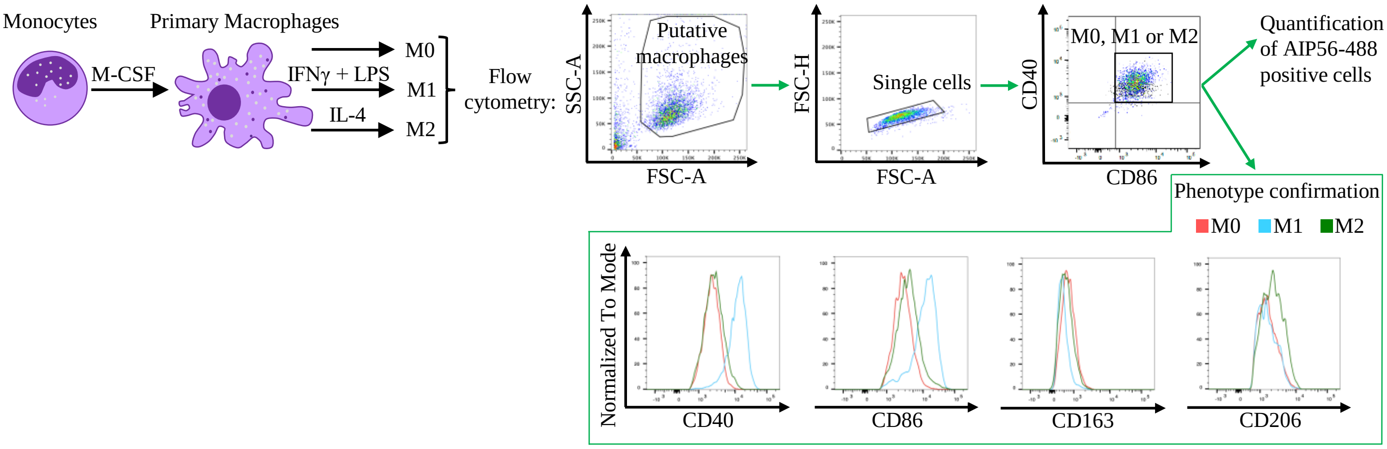

Figure S6. Schematic description of human monocytes differentiation into macrophages and their polarization. Representative plots of gating strategy applied to identify human macrophages, after selecting the general cell population through morphological parameters (SSC-A vs FSC-A) and eliminating duplets (FSC-H vs FSC-A). Macrophages subtypes (M0, M1-like and M2-like) were confirmed based on their expression of CD40, CD86, CD163 and CD206, respectively.

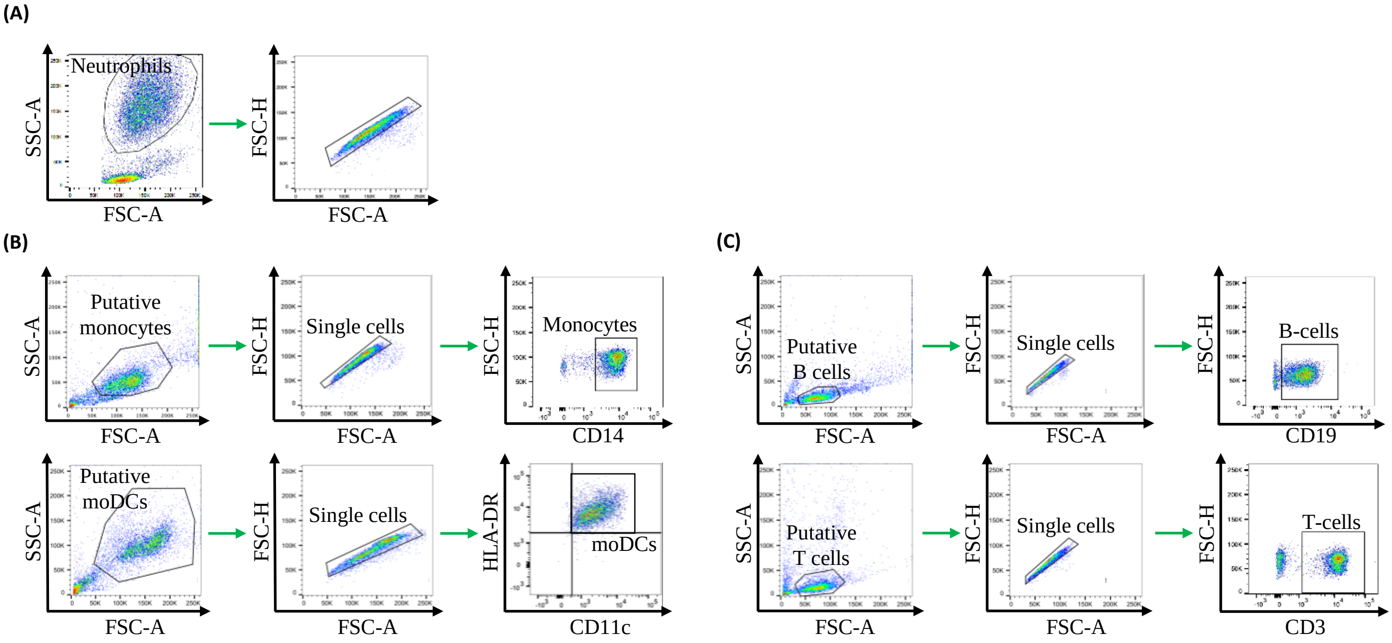

Figure S7. Gating strategy applied to identify different and viable human leukocytes. **(A)** Neutrophils were analyzed from whole peripheral blood, where neutrophils are the majority of the granulocytes. Neutrophils were gated based on morphological parameters (SSC-A vs FSC-A) and then, duplets were eliminated (FSC-H vs FSC-A). **(B)** Monocytes were isolated from buffy coats by MACS system, using CD14 beads and moDCs were derived from these cells. **(C)** The portion of cells that were negatively selected by CD14 beads were divided in B-cells and T-cells also by MACS system, using CD19 beads. **(B, C)** Gating strategy to identify the obtained monocytes, moDCs, B-cells and T-cells after selecting the general cell population through morphological parameters (SSC-A vs FSC-A) and eliminating duplets (FSC-H vs FSC-A).

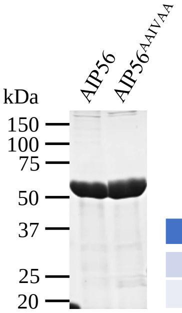

Protein	Purity
AIP56	93%
AIP56^{AAIVAA}	91%

Figure S8. SDS-PAGE analysis of purified recombinant proteins used in this study. Each lane was loaded with 10 μg of the indicated recombinant protein and the gel was stained with Coomassie Blue. Purity of the proteins was determined by densitometry using Image Lab software (Bio-Rad).
