# Supplementary material for: AIP56, an AB toxin secreted by Photobacterium damselae subsp. piscicida, has tropism for myeloid cells
Source: Front Immunol. 2025 Jan 13;15:1527088. doi: 10.3389/fimmu.2024.1527088 (PMC11769971; doi:10.3389/fimmu.2024.1527088)
Supplement: Supplementary file 3 [file Table1.pdf]

Table S1. AIP56-488 internalization by sea bass leukocytes.

Sea bass cell populations			% of fluorescent cells		MFI[1]
**Lineage**	**Source**	**Cell type**	**Untreated cells**	**AIP56-488 treated cells**	**AIP56-488 positive cells**
**Myeloid**	Peritoneum	Macrophages	0.06 ± 0.13	82.38 ± 9.44	46.83 ± 16.66
		Neutrophils	0.01 ± 0.02	13.40 ± 7.25	2.14 ± 0.90
**Lymphoid**	Spleen	IgM^+ Lymphocytes	0.67 ± 0.87	2.53 ± 0.43	1.36 ± 0.09
	Thymus	IgM^- Thymocytes	0.10 ± 0.07	0.58 ± 0.44	1.29 ± 0.19

[1]MFI (Median Fluorescence Intensity) results are presented as fold-change in fluorescence intensity of AIP56-488 positive cells relative to untreated cells (control). Values from each individual experiment are shown in Supplementary Material data sheet 1.

Table S2. AIP56-488 internalization by mouse leukocytes.

Mouse cell populations				% of fluorescent cells		MFI[1]
**Lineage**	**Source**	**Cell type**		**Untreated cells**	**AIP56-488 treated cells**	**AIP56-488 positive cells**
**Myeloid**	BM	Macrophages		0.31 ± 0.20	72.83 ± 9.86	6.47 ± 1.63
		Monocytes		0.82 ± 0.46	25.30 ± 3.29	2.12 ± 0.14
		Eosinophils		0.59 ± 0.56	3.87 ± 2.44	1.25 ± 0.12
		Neutrophils		0.18 ± 0.04	3.28 ± 1.17	1.16 ± 0.37
		mBMDM	M0	2.93 ± 0.48	23.50 ± 6.07	1.67 ± 0.23
			M1	2.08 ± 0.75	16.70 ± 2.33	2.07 ± 0.17
			M2	2.13 ± 0.69	36.60 ± 14.41	2.13 ± 0.41
	Spleen	Macrophages		0.38 ± 0.65	85.37 ± 2.57	8.86 ± 3.12
		Monocytes		1.11 ± 1.39	20.40 ± 4.13	2.24 ± 0.57
		DCs		0.00 ± 0.00	20.05 ± 6.45	3.32 ± 0.19
	Peritoneum	Macrophages		1.28 ± 0.19	98.30 ± 1.31	28.66 ± 9.41
		Neutrophils		0.28 ± 0.13	8.61 ± 1.72	0.95 ± 0.28
	Blood	Neutrophils		0.00 ± 0.01	7.00 ± 5.29	1.92 ± 0.18
**Lymphoid**	Spleen	B-cells		0.58 ± 0.96	8.17 ± 0.68	2.43 ± 0.88
		B1-cells		2.83 ± 3.68	33.85 ± 7.43	5.76 ± 0.64
		B2-cells		0.62 ± 0.74	10.89 ± 7.23	2.87 ± 0.41
		T-cells		0.09 ± 0.13	1.99 ± 0.99	1.19 ± 0.06
		Tc-cells		0.00 ± 0.00	1.02 ± 0.86	1.16 ± 0.09
		Th-cells		0.08 ± 0.08	1.11 ± 0.01	1.10 ± 0.02

[1]MFI (Median Fluorescence Intensity) results are presented as fold-change in fluorescence intensity of AIP56-488 positive cells relative to untreated cells (control). Values from each individual experiment are shown in Supplementary Material data sheet 1.

Table S3. AIP56-488 internalization by human leukocytes.

Human cell populations		% of fluorescent cells		MFI[1]
**Lineage**	**Cell type**	**Untreated cells**	**AIP56-488 treated cells**	**AIP56-488 positive cells**
**Myeloid**	M0	1.18 ± 0.76	91.87 ± 12.02	20.98 ± 4.17
	M1	0.48 ± 0.32	95.19 ± 4.16	7.66 ± 4.87
	M2	1.62 ± 1.18	96.62 ± 3.03	25.15 ± 10.87
	Monocytes	0.07 ± 0.06	93.57 ± 8.64	9.59 ± 4.44
	moDCs	0.47 ± 0.25	72.20 ± 15.57	5.30 ± 2.35
	Neutrophils	3.76 ± 6.04	61.10 ± 27.25	2.83 ± 0.91
**Lymphoid**	B-cells	0.16 ± 0.24	7.07 ± 0.85	1.50 ± 0.59
	T-cells	0.05 ± 0.03	1.10 ± 0.10	1.15 ± 0.10

[1]MFI (Median Fluorescence Intensity) results are presented as fold-change in fluorescence intensity of AIP56-488 positive cells relative to untreated cells (control). Values from each individual experiment are shown in Supplementary Material data sheet 1.
